# Efficacy of preoperative electroacupuncture for ureteral access sheath placement during first-stage flexible ureteroscopy in urolithiasis: a multicenter, randomized, single-blind, sham-controlled trial protocol

**DOI:** 10.1186/s12894-025-01993-3

**Published:** 2025-12-09

**Authors:** Shaoting Wang, Dexin Song, Xiangyang Zhan, Jinglan Hu, Dongliang Xu, Zubing Mei, Xinyu Zhai

**Affiliations:** 1 Urology Center, Shuguang Hospital, Shanghai University of Traditional Chinese Medicine, 528 Zhangheng Road, Pudong, Shanghai, 201203 China; 2 Surgical Institute of Integrative Medicine, Shuguang Hospital, Shanghai University of traditional Chinese Medicine, Shanghai, China; 3 Surgical Institute, Shuguang Hospital, Shanghai University of traditional Chinese Medicine, Shanghai, China; 4 Shanghai Key Laboratory of Traditional Chinese Clinical Medicine, Shanghai, China; 5 Department of Anorectal Surgery, Shuguang Hospital, Shanghai University of Traditional Chinese Medicine; Anorectal Disease Institute of Shuguang Hospital, 528 Zhangheng Road, Shanghai, 201203 China; 6 Anorectal Disease Institute of Shuguang Hospital, Shanghai, China

**Keywords:** Urolithiasis, Electroacupuncture (EA), Ureteroscopy, Ureteral access sheath, Randomized controlled trial

## Abstract

**Introduction:**

Retrograde intrarenal surgery (RIRS) is a primary treatment for urolithiasis, with successful ureteral access sheath (UAS) placement being a critical step. Preliminary studies suggest preoperative electroacupuncture (EA) may enhance UAS placement success, reduce ureteral injury, and improve stone clearance rates. This trial evaluates the efficacy and safety of EA as an adjunct to first-stage RIRS.

**Methods and analysis:**

This multicenter, randomized, single-blind, sham-controlled trial will enroll 120 adult patients with upper ureteral or renal stones (≥ 10 mm) scheduled for first-stage RIRS. Participants will be randomized (1:1) to receive either preoperative EA plus general anesthesia or sham EA plus general anesthesia across multiple sessions. The primary outcome is the proportion of patients with successful UAS placement during first-stage RIRS. Secondary outcomes include surgical duration, UAS insertion resistance, ureteral injury (Post-Ureteroscopic Lesion Scale [PULS]), stone clearance rate at 2 weeks post-surgery, and adverse events (AEs) up to 2 weeks post-surgery. Data will be analyzed using intention-to-treat principles.

**Discussion:**

This study is the first randomized controlled trial to investigate the efficacy of EA in improving the success rate and safety of UAS placement during first-stage RIRS. The findings will provide high-quality evidence to support the use of EA as an adjunctive therapy in clinical practice. Through a comprehensive multidimensional assessment, this study demonstrates the potential of EA for broader application in the treatment of urolithiasis. Further rigorously designed clinical trials are essential to validate and refine this promising therapeutic approach.

**Registration number:**

International Traditional Medicine Clinical Trial Registry. Identifier: ITMCTR2025001039.

**Supplementary Information:**

The online version contains supplementary material available at 10.1186/s12894-025-01993-3.

## Introduction

Urolithiasis remains a significant public health concern globally, with a particularly notable burden in China, where its prevalence is estimated at 7.54%, ranging from 1.61% to 20.45% across diverse regions [1]. Recent epidemiological surveys indicate an upward trend, driven by factors such as dietary shifts, dehydration, and metabolic disorders, making it one of the most common urological conditions encountered in clinical practice [2–4]. This rising incidence underscores the need for effective, minimally invasive treatments, particularly for upper ureteral and renal stones, which often require surgical intervention due to their size or location. RIRS has emerged as a preferred modality, leveraging flexible ureteroscopy and laser lithotripsy to fragment and remove stones with reduced morbidity compared to open surgery or percutaneous nephrolithotomy [5, 6]. Central to RIRS’s success is the placement of a UAS, a device that provides a stable conduit for the ureteroscope, facilitates irrigation, and aids in stone retrieval, thereby enhancing procedural efficiency and outcomes [7, 8].

Despite its advantages, UAS placement poses a critical technical challenge in RIRS, with failure rates reported in up to 25% of cases during primary procedures [9]. These failures stem primarily from ureteral muscle spasms triggered by stone obstruction and radial resistance due to anatomical stenosis, both of which impede smooth sheath insertion [10]. Violent manipulation to overcome such resistance can result in significant ureteral injuries—ranging from mucosal tears to perforation or rupture—complicating recovery and increasing postoperative morbidity [11]. However, the lack of quantitative data defining “excessive force” during UAS insertion complicates efforts to standardize safe practices. Moreover, studies have shown that UAS use, while not significantly altering stone-free rates or operative duration, elevates the risk of ureteral injury, positioning it as a procedural “double-edged sword” that demands careful management [12, 13]. These challenges highlight an urgent need for adjunctive strategies to improve UAS success rates, minimize trauma, and optimize patient outcomes in the context of RIRS.

Current approaches to enhance UAS placement include pharmacological interventions, notably preoperative alpha-blockers like silodosin, which relax ureteral smooth muscles and improve insertion success, achieving rates up to 82% in some trials [14]. This effect is attributed to their ability to reduce ureteral tone and spasm, yet their requirement for a 7-day pretreatment regimen poses a significant drawback, delaying surgery and prolonging patient discomfort—particularly problematic for those with acute pain or obstruction. Alternative studies have explored risk factors for UAS resistance, such as stone size, ureteral anatomy, and prior stenting, but these analyses often fail to propose actionable intraoperative solutions [15–17]. Mechanical aids, such as smaller-diameter sheaths or ureteral dilation, have been suggested, yet they carry their own risks, including increased injury or procedural complexity, and lack consistent evidence of efficacy [18–20]. Consequently, patients requiring urgent RIRS are left with limited options, emphasizing the need for a rapid, effective adjunct that can be deployed in the perioperative setting without systemic side effects or prolonged preparation.

EA, an advanced form by electrical stimulation, presents a compelling alternative. EA stimulates specific acupoints, offering therapeutic benefits across a spectrum of conditions, including pain and muscle spasms [21–23]. In addition, randomized controlled trials by Tu et al. [24, 25] also demonstrated the therapeutic efficacy of EA in pain management. Huang et al. [26] explored the analgesic effects of transcutaneous electrical nerve stimulation based on wrist-ankle acupuncture theory effectively reduced pain during colonoscopy without anesthesia, which suggests promising implications for EA in improving ureteral sheath placement outcomes, opening avenues for further research. A Systematic review by Saraogi M et al. [27] pointed out a decrease in analgesia or opiate use during the use of acupuncture with Shockwave Lithotripsy, a statistically significant lowered pain and/or anxiety score. A recent trial by Yang et al. [28] targeting diarrhea-predominant irritable bowel syndrome, with its robust methodology—including a blinded sham-controlled design and composite efficacy endpoints—provides a template for evaluating EA in urology. Our trial adapt to this model to assess the impact of EA on UAS placement outcomes. In the context of urolithiasis, EA’s potential lies in its multifaceted mechanisms: neural modulation via sensory afferent pathways activates parasympathetic reflexes and releases neurotransmitters like endorphins and acetylcholine, promoting ureteral relaxation [29, 30]; Endocrine effects, such as increased nitric oxide and prostaglandin synthesis, further reduce smooth muscle tone [31]; And local tissue improvements, including enhanced blood circulation and reduced edema, mitigate inflammation and spasm [32, 33]. Moreover, Jiang et al. [34] have demonstrated that EA commonly protects dopaminergic neurons by reducing neuroinflammation, oxidative stress, and apoptosis, based on these mechanisms, we think EA could also achieving the following therapeutic effects: alleviation of postoperative pain and edema, preservation of the ureteral epithelial barrier integrity, and facilitation of tissue repair. Wan et al. [35] indicated that the analgesic effect of EA in inflammatory and neuropathic pain is facilitated by CB1 receptor-mediated inhibition of GABA^RVM^ neurons. Preliminary experiments by our research group have demonstrated that preoperative EA may significantly increases UAS placement success, reduces ureteral injury, and enhances stone clearance rates during first-stage RIRS. These findings suggest EA could address the limitations of existing methods by providing an immediate, non-pharmacological intervention that aligns with the urgent needs of patients presenting with symptomatic stones.

This multicenter, randomized, single-blind, sham-controlled trial aims to rigorously evaluate the efficacy and safety of preoperative EA in improving UAS placement success during first-stage RIRS for urolithiasis. Building on our pilot data, which indicated a 90% UAS success rate with EA versus 75% in controls, we hypothesize that EA will outperform sham EA across key clinical endpoints. The primary objective is to assess the proportion of patients achieving successful UAS placement, defined as fluoroscopically confirmed insertion without alternative access methods. Secondary objectives include evaluating EA’s impact on surgical duration, UAS insertion resistance (measured objectively via a calibrated push-pull gauge), ureteral injury severity (assessed using the PULS), stone clearance rate at 2 weeks post-surgery, and adverse event profiles. By integrating EA into the RIRS workflow, this study investigates whether EA, as a safe and cost-effective adjunct, can reduce complications and residual stone rates after flexible ureteroscopy and accelerate patient recovery. Such findings could reshape clinical practice, offering a novel paradigm for managing urolithiasis in an era of rising prevalence and surgical demand.

## Methods

### Study design and objectives

This trial is a multicenter, randomized, single-blind, sham-controlled clinical study designed to assess the efficacy and safety of preoperative EA in improving UAS placement during first-stage RIRS for urolithiasis. It will be conducted across urology departments at Shuguang Hospital Affiliated with Shanghai University of Traditional Chinese Medicine and other collaborating hospitals in China. A single-blind approach is used because acupuncturists must know the intervention type to deliver EA or sham EA accurately, but patients, outcome assessors, and data analysts will remain unaware of group assignments to reduce bias. This design balances practical constraints with scientific integrity, drawing from established acupuncture trial methodologies [36]. The trial follows the Standard Protocol Items: Recommendations for Interventional Trials (SPIRIT) guidelines, with a detailed checklist provided in Supplementary File 1.

**Fig. 1:**
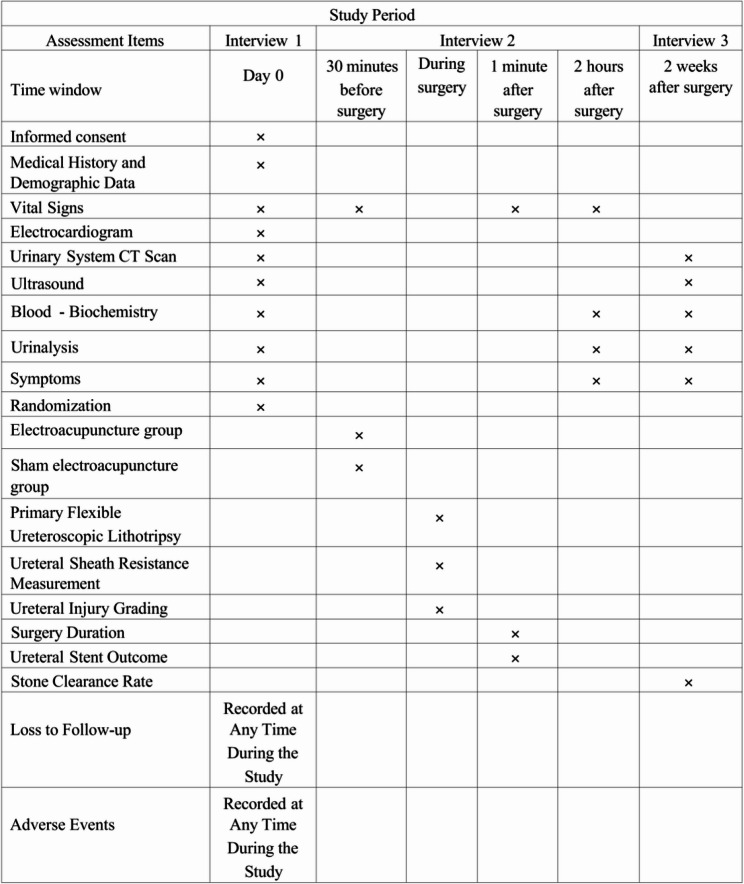
Clinical Study Flowchart

The study has clear objectives to guide its execution and analysis. The primary objective is to determine whether preoperative EA increases the proportion of successful UAS placements in first-stage RIRS compared to sham EA. Success is defined as the ability to insert the UAS and complete the procedure without needing alternative access methods, confirmed by fluoroscopy. Secondary objectives include evaluating EA’s effects on surgical duration, resistance during UAS insertion, degree of ureteral injury at the end of surgery, stone clearance rate two weeks after surgery, and the occurrence of adverse events (AEs) during and up to two weeks post-surgery. These endpoints aim to provide a comprehensive picture of EA’s impact on procedural efficiency, safety, and patient recovery. By focusing on both immediate surgical outcomes and short-term postoperative results, the trial seeks to offer practical insights for urologists managing urolithiasis. The multicenter setting enhances the generalizability of findings, while the sham control helps isolate EA’s specific effects from placebo responses, ensuring robust evidence for clinical decision-making. The schedule of enrollment, intervention and assessment is displayed in Fig. 1. Beyond the predefined week-2 assessment, an exploratory clinic visit at week-12 will capture symptoms, urinalysis, and ultrasound; CT urography will be obtained if clinically indicated to screen for delayed ureteral strictures. Delayed complications and reinterventions up to week-12 will be descriptively reported.

### Inclusion and exclusion criteria

Participants will be carefully selected to ensure the trial targets the appropriate population while maintaining safety and feasibility. Inclusion criteria are as follows: Patients aged 18 to 75 years (including both 18 and 75), diagnosed with upper ureteral or renal stones measuring 10 mm or larger via urinary system computed tomography (CT), scheduled for first-stage RIRS, and able to provide written informed consent. These criteria focus on adults with stones requiring RIRS, a common clinical scenario where UAS placement is critical. The age range accommodates a broad adult population while excluding extremes that might complicate anesthesia or acupuncture. Informed consent ensures participants understand the study’s purpose, procedures, risks, and benefits, aligning with ethical standards outlined in the Declaration of Helsinki.

Exclusion criteria are designed to eliminate factors that could confound results or pose risks: patients with active urinary or reproductive system infections or fever, those who used alpha-blockers within the past 4 weeks, individuals with a history of surgery on a solitary kidney or congenital/acquired ureteral or urethral anomalies, those afraid of needles, participants in other interventional studies within 3 months, individuals with severe psychological or cognitive impairments affecting consent or compliance, and those with known allergies to acupuncture needles. These exclusions address potential biases (e.g., prior alpha-blocker use altering ureteral tone), safety concerns (e.g., infections or allergies), and procedural challenges (e.g., anatomical anomalies complicating UAS placement). By setting these boundaries, the trial ensures a homogeneous study group, reduces variability in outcomes, and protects participant well-being, consistent with rigorous clinical research standards.

### Recruitment and randomization

**Fig. 2:**
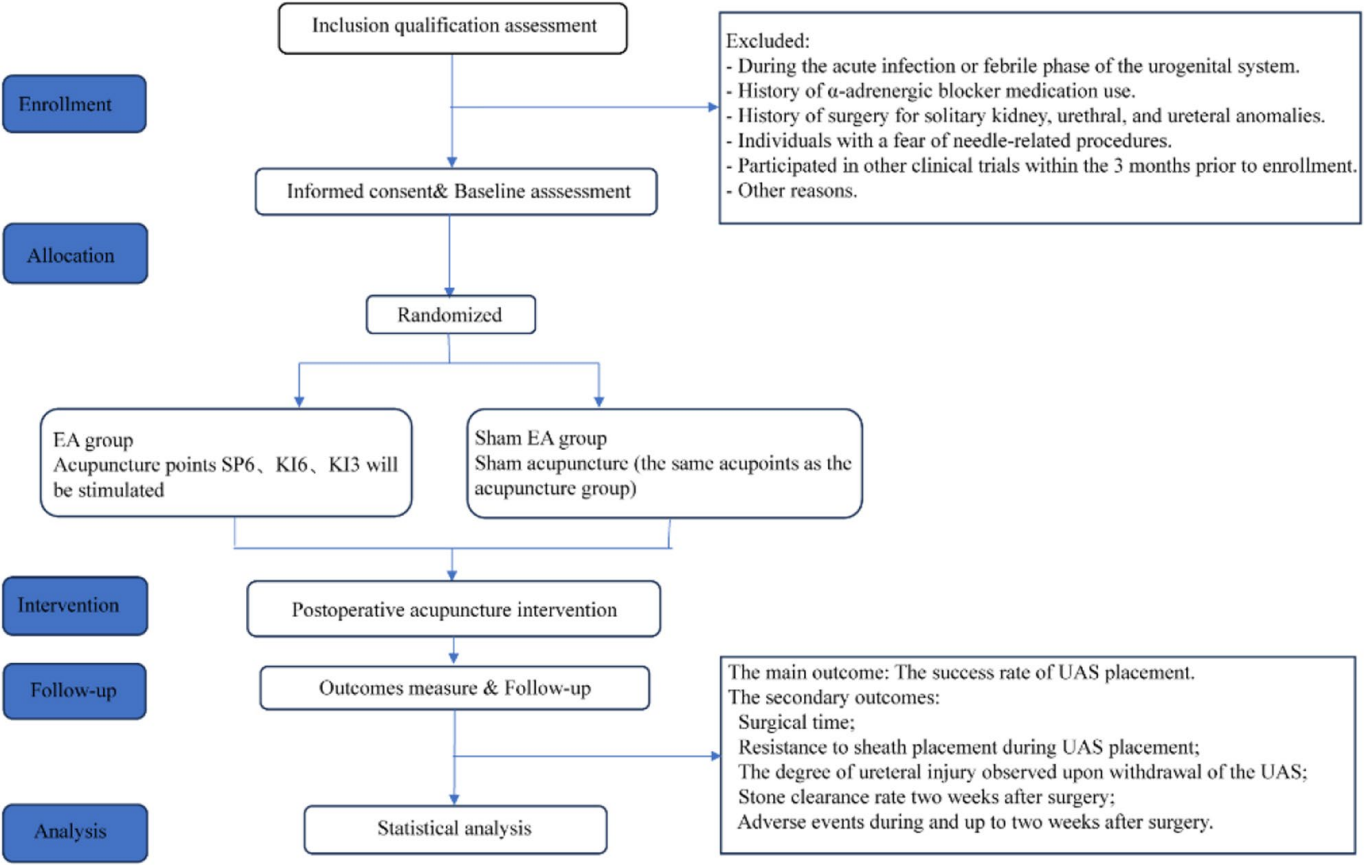
Trial flow chart. Abbreviations: EA, Electroacupuncture; UAS, Ureteroscope access sheath

Recruitment will occur at the urology departments of participating hospitals, targeting patients scheduled for first-stage RIRS, period is anticipated to span six months, from 15 Aug 2025 to 16 Feb 2026. Eligible patients will be identified through hospital wards, approached by research staff, and provided with detailed study information, including a consent form explaining the trial’s purpose, procedures, and voluntary nature. This process ensures transparency and allows patients to ask questions before agreeing to participate. Recruitment will continue until the target sample size of 120 is reached, with efforts to maintain a steady enrollment pace across centers to avoid seasonal or site-specific biases. The flow chart of study protocol is described in Fig. 2.

Randomization will assign participants to either the EA or sham EA group in a 1:1 ratio using a computer-generated random number sequence created with SAS software (version 9.4) by an independent statistician not involved in patient care or data collection. To prevent selection bias, allocation concealment will be achieved using sequentially numbered, opaque envelopes prepared by the statistician and opened by a trial coordinator only after a patient signs the consent form, following CONSORT guideline [37]. This method ensures that neither recruiters nor participants can predict or influence group assignments. The randomization sequence will be stratified by center to account for potential differences in surgical practices or patient demographics across sites. Envelopes will be stored securely, and their use documented to maintain traceability, reinforcing the trial’s fairness and objectivity.

### Interventions

**Fig. 3:**
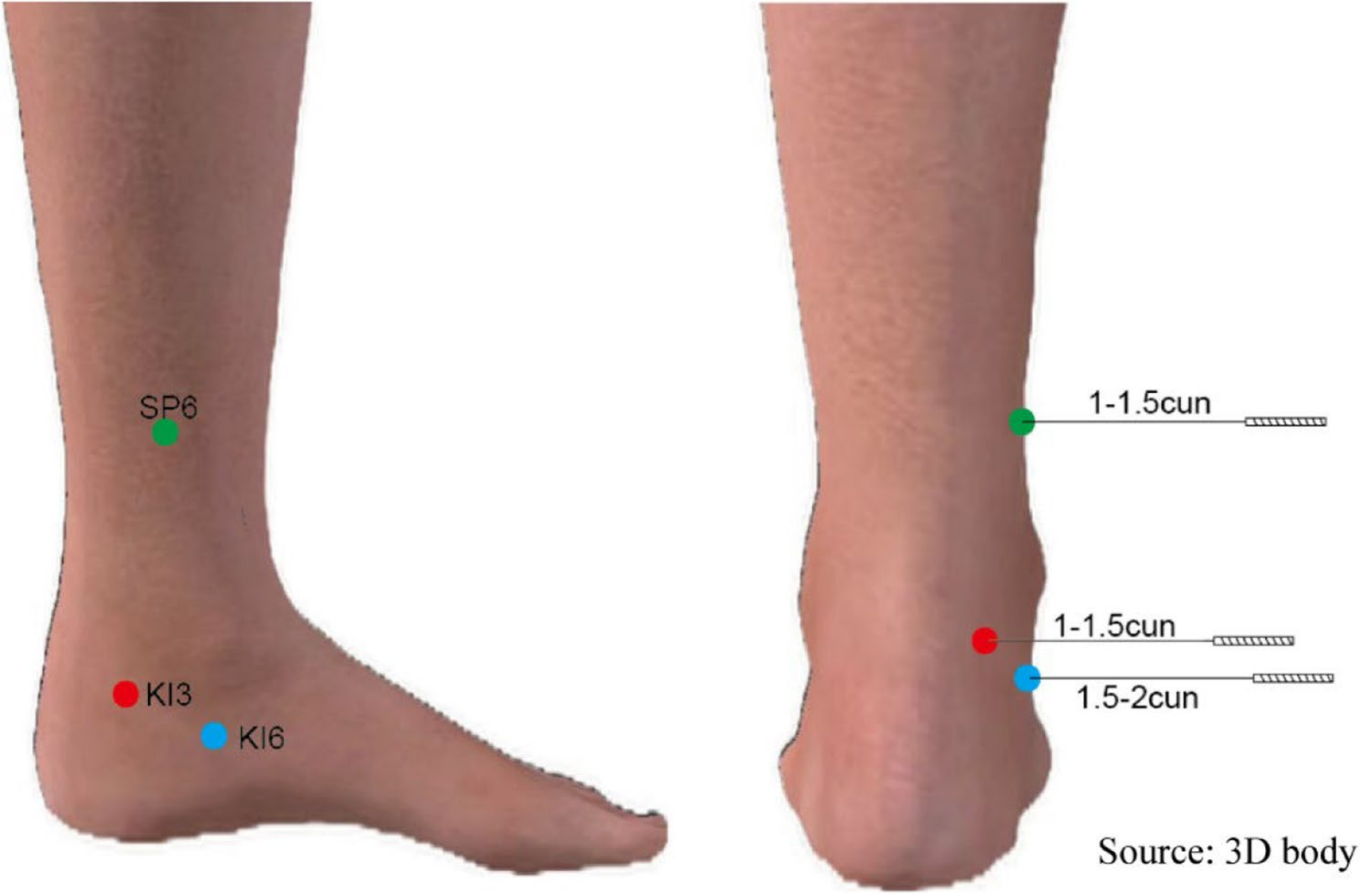
Illustration of Acupuncture Point Locations

Both groups will undergo first-stage RIRS under general anesthesia; no routine preoperative ureteral stenting will be performed. Ureteral stents will be placed only as intraoperative rescue (e.g., failed UAS placement or ureteral injury) at the surgeon’s discretion. EA will start 30 min before anesthesia induction and continue at least until completion of the first UAS attempt; total stimulation time will be recorded. A harmonized anesthetic pathway will be used across centers as detailed in the peri-anesthetic standardization section. Licensed acupuncturists with at least 5 years of experience will insert sterile needles (0.25 mm × 40 mm, Huatuo brand) into Sanyinjiao (SP6), Zhaohai (KI6), and Taixi (KI3) bilaterally. After a 30-second lifting-thrusting technique, needles will be connected to an EA device (SDZ-II, Huatuo) delivering a 50 Hz continuous wave at 1–5 mA, adjusted to the patient’s maximum tolerable level without pain. Acupuncturists will be trained beforehand to standardize needle insertion and stimulation, with records kept of each session’s completion. If acupuncture-related AEs occur (e.g., bleeding), needles will be removed promptly. All participants will receive standardized perioperative care, including general anesthesia, postoperative analgesia, and prophylactic antibiotics. Use of α-blockers, calcium channel blockers, or other medications affecting ureteral tone was prohibited. Compliance was monitored through medication history reviews and patient records. The locations of acupoints are illustrated in Fig. 3, with precise needling positions and procedural details summarized in Table 1.

**Table 1:** Precise needling positions and procedural details

Acupoint (international code)	Location	Angle and Depth
Sanyinjiao(SP6)	The medial side of the lower leg, 3 cun above the tip of the medial malleolus, posterior to the medial border of the tibia.	Insert the needle perpendicularly to the skin to a depth of 1–1.5.5 cun.
Zhaohai (KI6)	1 finger-width medial to the ankle joint.	Insert the needle perpendicularly to the skin to a depth of 1.5–2.5 cun.
Taixi (KI3)	2 finger-width medial to the ankle joint.	Insert the needle perpendicularly to the skin to a depth of 1–1.5.5 cun.

Acupoint nomenclature follows the WHO Standard Acupuncture Point Locations (2008). *Abbreviations* *SP* Spleen Meridian, *KI* Kidney Meridian

The sham EA group will receive a placebo intervention mimicking EA: needles will be inserted superficially (1–4 mm) 3 mm lateral to the true acupoints without manipulation or electrical current. The EA device will be connected but internally short-circuited, producing sounds and lights to maintain blinding. Patients will be told they might receive either real or sham treatment, and acupuncturist interactions will follow a script to avoid revealing group assignments. This sham design, adapted from prior acupuncture trials, ensures participants remain blinded while minimizing therapeutic effects. Both interventions will be performed by trained acupuncturists, with blinding success assessed post-surgery via patient surveys asking them to guess their group. The similarities and differences in acupuncture procedures between the experimental and control groups are detailed in Table 2.

**Table 2:** Operational similarities and differences between the experimental and control groups

Program	Experimental Group	Control Group
Common Points		
Stimulated Point Area	Leg	
Number of Acupoints/Non-Acupoints	6	
Number of Needles	6	
Specifications of Needling Instruments	Length: 4 mm, Diameter: 0.25 mm	
Difference		
Types of Stimulated Points	Acupoint	Non-Acupoint
Location of Stimulated Points	Sanyinjiao (SP6), Zhaohai (KI6), Taixi (KI3)	3 mm lateral to the acupoints in the experimental group
Needling Depth	Approximately 10–30 mm	1–4 mm
Deqi Sensation	Yes	No
Actual Current Output	Yes	No

Acupoint nomenclature follows the WHO Standard Acupuncture Point Locations (2008). *Abbreviations* *SP* Spleen Meridian, *KI* Kidney Meridian

### Peri-anesthetic standardization

EA will start 30 min before anesthesia induction and continue at least until completion of the first UAS attempt; total stimulation time will be recorded. A harmonized anesthetic pathway will be used across centers: induction with propofol (1.5–2.5 mg/kg) plus remifentanil (TCI 2–4 ng/mL) or fentanyl (1–2 µg/kg), and rocuronium (0.6 mg/kg); maintenance with either TIVA (propofol/remifentanil) or sevoflurane (0.8–1.2 MAC). The first UAS attempt will occur after intubation and positioning but before any intraureteral dilators or antispasmodics are administered.

### Operator qualification and inter-rater agreement

Primary surgeons must each have performed ≥ 100 ureteroscopies. A site-initiation training will standardize surgical SOPs and PULS grading. Dual, blinded PULS ratings will be obtained centrally; inter-rater reliability will be summarized with weighted κ. Surgeon experience (years in practice; annual URS volume) will be recorded and considered in adjusted analyses.

### Operative standardization and covariates

A harmonized intraoperative framework will be used: preferred UAS size 12/14F; if downsizing to 11/13F is chosen, the rationale must be documented. Irrigation will target 40 cmH₂O (gravity) or 40 mmHg (pump). Stone burden (cumulative stone diameter and volumetry) and Hounsfield units will be extracted from preoperative CT; relevant anatomic features will be captured on a standardized form. These variables will be prespecified covariates in adjusted analyses.

### Safety monitoring and AE grading

EA-related AEs will be graded per CTCAE v5.0, while surgical complications will be graded per Clavien–Dindo. Serious AEs (death, life-threat, hospitalization, disability, or events requiring intervention) will be reported to the coordinating center and IRBs within 24–72 h as applicable.

### Study outcomes

The trial’s outcomes are chosen to evaluate EA’s impact comprehensively. The primary outcome is the proportion of patients with successful UAS placement in first-stage RIRS, defined as sheath insertion confirmed by fluoroscopy without requiring alternative access methods like dilation or a smaller sheath.

Secondary outcomes include: (1) Surgical duration, recorded in minutes from incision to closure; (2) UAS insertion resistance, measured with an IMADA50N push-pull gauge (calibrated monthly for accuracy); (3) Ureteral injury degree, assessed at UAS removal using the PULS by two blinded urologists, with a third resolving discrepancies; (4) Stone clearance rate, defined as the proportion of patients stone-free on CT two weeks post-surgery; and (5) AEs, tracked during surgery and for two weeks afterward, including acupuncture-related (e.g., bruising) and surgical (e.g., infection) events. These outcomes capture procedural efficiency, tissue safety, and recovery, with standardized tools and blinded assessments ensuring reliability. Training on PULS grading will be provided to assessors to maintain consistency.

### Outcome assessment and blinding

Key fluoroscopic snapshots and 15–30-s endoscopic clips of UAS insertion/removal will be uploaded to a central repository. Two independent urologists, blinded to allocation and site, will adjudicate UAS success and PULS; disagreements will be resolved by a third adjudicator. Blinding success will be quantified using Bang’s Blinding Index within 24–48 h after surgery.

### Statistical considerations

#### Estimation sample size

The sample size is based on data showing a 75% [9] UAS success rate in controls, and we hypothesized an 90% success rate for EA in a larger sample size. Using a two-sided chi-square test with a significance level of 0.05 and 0.28 confidence interval width, 55 patients per group (110 total) are needed to detect this difference. Accounting for a 10% dropout rate—due to withdrawal, incomplete follow-up, or protocol deviations—the sample size increases to 120 (60 per group), calculated using PASS software (version 2023) with parameters: two independent proportions module, alpha = 0.05, confidence = 0.95, precision = 0.28, EA success = 90%, sham success = 75%. This ensures sufficient power to detect a clinically meaningful effect while accommodating real-world challenges.

#### Statistical analysis

 All analyses will use the intention-to-treat (ITT) principle, including all randomized patients in their assigned groups regardless of adherence. Primary analysis will use a generalized linear mixed model (logit link) with study center as a random intercept and treatment group as a fixed effect (OR, 95% CI). Unadjusted χ²/Fisher’s exact tests will also be reported. Multiplicity across secondary endpoints will be controlled using the Benjamini–Hochberg procedure with a false discovery rate of 5%. Ordinal outcomes (e.g., PULS) will be modeled via proportional-odds regression. Missing data will be handled by multiple imputation (MICE, m = 20, MAR), with sensitivity analyses including complete-case, worst-case imputation, and a tipping-point analysis. Prespecified covariates: age, sex, stone burden (cumulative stone diameter or volumetry), stone density (HU), prestenting, UAS size, irrigation pressure, and anesthetic modality. Sample size: assuming 75% vs. 90% UAS success (control vs. EA), α = 0.05 and 80% power require 55 patients per group; allowing 10% attrition yields a total of 120.

#### Data collection and quality management

 Data will be collected using standardized electronic case report forms (e-CRFs) completed by trained staff during surgery and follow-up visits. Key variables—UAS success, surgical duration, insertion resistance, ureteral injury, stone clearance, and AEs—will be recorded in real time or at specified intervals (e.g., CT at 2 weeks). If participants withdraw early, collected surgical data are retained and followed by brief remote follow-ups. To ensure accuracy, data will be double-entered into a secure electronic database, with discrepancies resolved by checking original records like surgical notes or imaging reports. Blinding success will be evaluated post-surgery by asking patients to guess their group, aiming for a correct guess rate near 50% (chance level). Force measurements (IMADA50N) will follow manufacturer guidelines, with quarterly audits of calibration logs, retained for 10 years post-trial.Quarterly audits will verify gauge calibration logs and completeness of image/video uploads; all adjudication materials will be retained for 10 years.

Quality will be maintained through Good Clinical Practice (GCP) guidelines, with a data management plan outlining cleaning, coding, and storage procedures. De-identified data will be stored securely for 10 years and made available via a public repository (e.g., ClinicalTrials.gov) after publication, promoting transparency. Regular monitoring by the research team will ensure protocol adherence and data integrity across centers.

#### Ethics and dissemination

The trial has been approved by the Ethics Committee of Shuguang Hospital (2025-1773-113-02) and complies with the Declaration of Helsinki, China’s 2016 Biomedical Research Ethics Measures, and GCP guidelines. Before enrollment, patients will receive detailed oral and written information about the study, including its purpose, procedures, risks (e.g., minor bruising from needles), benefits, and their right to withdraw at any time without explanation. Written consent will be obtained, and patients will allow access to prior medical records for baseline data. Participants’ privacy will be protected by de-identifying data, with personal details like names and contact information kept confidential.

Results will be disseminated through peer-reviewed journals and presentations at national and international conferences, regardless of findings, to inform clinical practice and future research. De-identified datasets will be deposited in a repository upon completion, ensuring accessibility while safeguarding participant anonymity. This approach upholds ethical standards and maximizes the trial’s impact on urolithiasis management.

## Discussion

### Principal findings

This multicenter, randomized, single-blind, sham-controlled trial is designed to be the first to assess the efficacy of preoperative EA in improving UAS placement during first-stage RIRS for urolithiasis. The previously cited 15% absolute difference (90% vs. 75%) reflects pilot data used to inform the sample-size calculation; the present protocol is designed to estimate the treatment effect with appropriate precision rather than to assume such benefit a priori. Secondary outcomes will be evaluated as prespecified endpoints (operative time, insertion resistance, PULS grade, and two-week stone-free status), with their interpretation framed as exploratory and supportive of the primary hypothesis. Safety monitoring will follow standardized definitions and windows (e.g., CTCAE/Clavien–Dindo) without presuming acupuncture-related event rates. These outcomes address a critical challenge in RIRS, where UAS placement failures and associated injuries remain significant hurdles. If efficacy is demonstrated, this trial could position EA as a practical, non-invasive adjunct, enhancing procedural efficiency and patient recovery in urological practice.

### Potential mechanisms

EA’s potential to improve UAS placement likely arises from its multifaceted effects on ureteral smooth muscle relaxation, as supported by preclinical and pilot studies. Neural mechanisms involve stimulation of acupoints like Sanyinjiao (SP6), Zhaohai (KI6), and Taixi (KI3), activating sensory afferents and parasympathetic pathways that release endorphins and acetylcholine to reduce ureteral spasm. Additionally, endocrine effects may increase nitric oxide and prostaglandin production, lowering ureteral tone and aiding dilation during sheath insertion. Locally, EA enhances blood flow, reducing edema and inflammation, which could decrease resistance and protect ureteral tissue from trauma. Within this protocol, these pathways are treated as hypotheses; where feasible, ancillary measures will contextualize (but not test) mechanistic plausibility.

### Comparisons with similar studies

This trial’s focus on preoperative EA for UAS placement distinguishes it from related urolithiasis studies, providing a novel intraoperative perspective. Zhang et al. [10] combined EA with extracorporeal shock wave lithotripsy (ESWL) to enhance postoperative stone expulsion, reporting improved clearance rates but not addressing UAS placement directly; our study targets immediate procedural success rather than post-treatment outcomes. Diab et al. [14] demonstrated an 82% UAS success rate using preoperative alpha-blockers (e.g., silodosin) over 7 days, yet this approach delays surgery and risks side effects like hypotension, unlike EA’s immediate, drug-free application. A meta-analysis by Fahmy, Omar et al. [38] found preoperative stenting increased UAS success but required an additional procedure, contrasting with EA’s single-session delivery. Wen et al. [39] explored UAS-related ureteral injuries, noting higher PULS grades with forceful insertion, but offered no preventive strategies; our trial tests EA as a proactive solution. These comparisons underscore our study’s unique contribution: leveraging EA to improve RIRS efficiency and safety without pretreatment delays or additional interventions, while prospectively evaluating outcomes in a randomized, sham-controlled framework.

### Strengths

The trial’s design and execution offer several key strengths. First, its multicenter approach across Chinese hospitals ensures a diverse patient sample, enhancing the generalizability of results to varied clinical settings. Second, randomization and a sham-controlled structure isolate EA’s specific effects from placebo responses, strengthening causal inference. Third, objective outcome measures—such as fluoroscopy-confirmed UAS success, IMADA50N force gauge readings, and PULS grading by blinded assessors—provide reliable, reproducible data. Fourth, outcomes will undergo central blinded adjudication with predefined criteria, and a blinding assessment (e.g., Bang’s Blinding Index) will quantify the success of patient blinding. Fifth, analyses will follow an intention-to-treat framework with prespecified sensitivity analyses to assess robustness. Sixth, standardized acupuncturist training and scripted interactions across sites aim to reduce performance variability and enhance intervention fidelity.

### Limitations

Despite these strengths, the trial has notable limitations. First, the single-blind design, where acupuncturists are unblinded, risks performance bias, though standardized procedures aim to mitigate this. Second, the sample size of 120 is powered for the primary outcome but may lack sufficient power to detect differences in secondary endpoints like stone clearance, limiting conclusive interpretations. Third, multiplicity across secondary endpoints will be addressed using a prespecified approach (e.g., false-discovery-rate control), yet these analyses remain exploratory. Fourth, the study’s focus on Chinese patients may restrict applicability to other ethnic groups with differing anatomical or physiological profile. Fifth, the short follow-up (two weeks) precludes assessment of long-term outcomes like ureteral stricture rates. To partially address this, an exploratory week-12 follow-up has been incorporated to descriptively capture delayed complications and reinterventions.

### Clinical practice value and future directions

A successful trial could integrate EA into RIRS protocols, offering significant clinical benefits. If the primary hypothesis is supported, EA’s immediate, session-based application may reduce reliance on pretreatment strategies that delay surgery (e.g., alpha-blockers) or add procedures (e.g., routine pre-stenting), with potential advantages in tolerability and cost [14]. Future research should refine stimulation parameters (frequency, acupoint sets), incorporate physiologic biomarkers (e.g., ureteral pressure or inflammatory markers) to strengthen mechanistic inference, and power key secondary endpoints in larger, possibly international cohorts. Evaluating EA in related endourologic scenarios (e.g., primary stent placement or difficult ureteral access) could broaden its scope if efficacy and safety are confirmed.

## Conclusions

This protocol will deliver randomized, sham-controlled evidence regarding the effect of preoperative EA on UAS placement success in first-stage RIRS. Findings will inform clinical practice if efficacy is demonstrated and will guide the design of future trials aimed at optimizing perioperative adjuncts in endourology.

## Supplementary Information

Supplementary Material 1: S1 File. SPIRIT checklist.

Supplementary Material 2: S2 File. Study protocol approved by the ethics committee (in English).

Supplementary Material 3: S3 File. Study protocol approved by the ethics committee (in Chinese).

Supplementary Material 4: Human Subjects Research Checklist.

## Data Availability

No datasets were generated or analysed during the current study. All relevant data from this study will be made available upon study completion.

## Abbreviations

AEs: Adverse events
CT: Computed tomography
EA: Electroacupuncture
E-CRFs: Electronic case report forms
ESWL: Extracorporeal shock wave lithotripsy
GCP: Good clinical practice
HZ: Hertz
ITT: Intention-to-treat
KI: Kidney meridian
PULS: Post-Ureteroscopic lesion scale
RIRS: Retrograde intrarenal surgery
SAS: Statistical analysis system
SP: Spleen meridian
UAS: Ureteroscope access sheath
