# Supplementary material for: Efficacy of preoperative electroacupuncture for ureteral access sheath placement during first-stage flexible ureteroscopy in urolithiasis: a multicenter, randomized, single-blind, sham-controlled trial protocol
Source: BMC Urol. 2025 Dec 9;25:301. doi: 10.1186/s12894-025-01993-3 (PMC12690886; doi:10.1186/s12894-025-01993-3)
Supplement: Supplementary file 2 — Supplementary Material 2: S2 File. Study protocol approved by the ethics committee (in English). [file 12894_2025_1993_MOESM2_ESM.pdf]

Efficacy Evaluation of Electroacupuncture on the Success Rate of Ureteral Access Sheath Placement During first-stage Flexible Ureteroscopic Lithotripsy: A Randomized, Single-Blind, Sham-Controlled, Parallel-Group, Multicenter Clinical Trial

Principle Investigator: Prof. Xy Zhai, professor of the Department of Urological Surgery, Shuguang Hospital, Shanghai University of Traditional Chinese Medicine.

E-mail: zhaixinyu_2008@sina.com

Address:528 Zhangheng Road, Shanghai 201203, China

Version: 2.0

Date: April 20, 2025

Abstract

Introduction: Retrograde intrarenal surgery (RIRS) is a primary treatment modality for urinary tract stones, and successful placement of the ureteral access sheath (UAS) is a critical procedural step. Preliminary studies have suggested that preoperative electroacupuncture (EA) may improve UAS placement success rates, reduce ureteral injury, and enhance stone clearance outcomes. This trial evaluates the efficacy and safety of EA as an adjunctive therapy in first-stage RIRS.

Methods and analysis: This multicenter, randomized, single-blind, sham-controlled trial will enroll 120 adult patients with upper ureteral or renal stones (≥10 mm) scheduled for first-stage RIRS. Participants were randomly allocated (1:1) to receive either preoperative EA combined with general anesthesia or sham EA combined with general anesthesia across multiple treatment sessions. The primary outcome is the proportion of patients achieving successful UAS placement during first-stage RIRS. Secondary outcomes included operative time, resistance during UAS insertion, ureteral injury (assessed via the Post-Ureteroscopic Lesion Scale [PULS]), stone clearance rate at 2 weeks postoperatively, and adverse events (AEs) within 2 weeks postoperatively. Data will analyzed using the intention-to-treat principle.

Discussion: This study will be the first randomized controlled trial (RCT) to investigate the efficacy of EA in improving both the success rate and safety of UAS placement during first-stage RIRS. The findings will provide high-quality evidence to support the integration of EA as an adjunctive therapy in clinical practice. Through comprehensive multidimensional assessments, this research will demonstrate the potential for broader application of EA in urolithiasis management. Further rigorously designed clinical trials will be essential to validate and refine this promising therapeutic approach.

Study Protocol

1.Study background

1. RIRS will serve as a cornerstone intervention for urinary tract calculi, with the successful placement of the UAS during the procedure being a pivotal step. Identifying strategies to effectively improve UAS deployment success rates will be critical.

Urolithiasis will remain a prevalent condition in urology, with recent epidemiological surveys projecting a prevalence rate of 1.61%–20.45% in China, an

aggregate rate of 7.54%, and a rising trend [1]. Minimally invasive endoscopic procedures, including rigid ureteroscopy, flexible ureteroscopy, percutaneous nephrolithotomy, and laparoscopic lithotomy, will be required for many patients [2]. Advancements in technology will establish RIRS as a primary modality for urinary stone management. During RIRS, the placement of the UAS will constitute the initial and crucial procedural step [3]. Successful UAS insertion will depend on mitigating resistance caused by ureteral spasm secondary to stone obstruction and inherent ureteral strictures. Excessive force during sheath placement will risk ureteral injury, including perforation, avulsion, or rupture [3]. However, quantitative thresholds defining "excessive force" will remain undefined. While studies will identify risk factors influencing insertion resistance [4–6], effective protocols to reduce resistance and ureteral trauma during first-stage RIRS will not yet be established. Preoperative alpha-blocker therapy administered one week prior to surgery will enhance UAS placement success rates in first-stage RIRS. However, patients presenting with acute pain may find a one-week medication delay incompatible with their urgent need for symptom relief. Thus, developing approaches to expedite surgery while optimizing UAS insertion success will be imperative.

2.EA—demonstrating potential to relax ureteral smooth muscle effectively. However, whether EA can enhance the success rate of UAS placement during first-stage RIRS will remain unexplored.

Clinically, acupuncture will exhibit efficacy across diverse conditions: alleviating pain (e.g., headaches, musculoskeletal pain, arthritis), treating neurological disorders (e.g., facial paralysis, post-stroke sequelae, insomnia), and modulating gastrointestinal function (e.g., gastralgia, diarrhea, constipation). It will also address gynecological, andrological, and otorhinolaryngological ailments. Compared to modern medicine,

acupuncture will retain unique advantages, including simplicity, cost-effectiveness, and minimal adverse effects, often yielding unexpected benefits for chronic and refractory diseases. Furthermore, its integration with therapies such as electrical stimulation will enhance therapeutic outcomes.

EA, an adjunctive therapy integrating acupuncture with modern medicine, has demonstrated significant clinical efficacy in alleviating various types of muscle spasms and pain [5–6]. Research indicates that EA exerts a multifaceted relaxant effect on ureteral smooth muscle, primarily through the following mechanisms:

2.1 Neuromodulatory Mechanisms

2.1.1 Neural Pathway Activation: EA stimulates specific acupoints, activating sensory afferent pathways of the nervous system. These signals are transmitted to the central nervous system (CNS), triggering neural reflexes that modulate autonomic nervous system (ANS) activity. By regulating the balance between sympathetic and parasympathetic activity, EA influences ureteral smooth muscle tone. Parasympathetic excitation induces ureteral smooth muscle relaxation, facilitating urine drainage, whereas sympathetic activation may promote contraction.

2.1.2 Neurotransmitter Release: EA stimulation promotes the release of neurotransmitters such as endorphins and acetylcholine (ACh). Endorphins exert analgesic effects and directly relax smooth muscle, including the ureter. Acetylcholine modulates ureteral contraction and relaxation under certain conditions, suggesting that EA may regulate ureteral function via ACh-mediated pathways.

2.2 Endocrine Regulatory Mechanisms

2.2.1 Hormonal Modulation: EA may modulate the endocrine system to influence hormone secretion and release, thereby affecting ureteral smooth muscle function. Key mediators such as prostaglandins and nitric oxide (NO) play critical roles in

ureteral motility regulation. EA may adjust the synthesis and release of these hormones, altering ureteral smooth muscle tone.

2.2.2 Fluid-Electrolyte Balance: The endocrine system also regulates fluid-electrolyte balance, indirectly impacting ureteral function. By modulating renal water and electrolyte excretion, EA may influence urine production and flow, subsequently affecting ureteral pressure and peristalsis.

2.3 Local Tissue Regulatory Mechanisms

2.3.1 Enhanced Blood Circulation: EA promotes local blood circulation, improving oxygen and nutrient delivery while reducing the accumulation of metabolic byproducts. This mitigates tissue edema and inflammation, alleviating ureteral smooth muscle spasm.

2.3.2 Muscle Tone Regulation: EA may directly regulate ureteral smooth muscle tone. Stimulation of specific acupoints induces alternating contraction and relaxation of local muscles, modulating ureteral tension through neural reflexes, humoral regulation, or direct mechanical stimulation.

The mechanisms underlying EA-induced ureteral smooth muscle relaxation are multifaceted, involving neuroendocrine and local tissue regulation. While research into these mechanisms remains ongoing, EA—a fusion of traditional and modern therapeutic approaches—has demonstrated clinical efficacy in relieving ureteral spasms and enhancing urinary drainage.

3. Preliminary experiments conducted by our research team have demonstrated that EA significantly enhances the success rate of UAS placement, reduces ureteral injury, and improves stone clearance rates during first-stage RIRS, warranting further investigation.

Preliminary investigations by our research team have demonstrated that EA significantly enhances the success rate of UAS placement, mitigates intraoperative ureteral injury, and improves stone clearance rates during first-stage RIRS. The observed efficacy may be attributed to EA's multifaceted mechanisms. First, optimizing UAS placement is critical to procedural success, as it serves as the foundational step in RIRS. By modulating ureteral smooth muscle tone, EA likely induces relaxation, thereby facilitating sheath insertion, reducing operative time and technical challenges, and enhancing overall procedural safety. Second, EA's ability to minimize ureteral trauma addresses a major intraoperative risk. Ureteral injury during RIRS may lead to postoperative complications such as strictures or urinary leakage. EA appears to exert protective effects by improving local microcirculation and attenuating inflammatory responses, thereby preserving tissue integrity. Finally, improved stone clearance is vital for patient recovery, as residual calculi increase recurrence risks and impose physical and economic burdens. EA may enhance intraoperative stone expulsion by promoting ureteral peristalsis and optimizing urinary drainage.

In-depth exploration of the mechanisms underlying EA in RIRS could yield novel therapeutic strategies for clinical practice. Future studies should prioritize optimizing EA parameters (e.g., stimulation intensity, frequency) and acupoint selection to maximize efficacy and safety. Integrating advanced medical technologies, such as real-time imaging and physiological monitoring, may further elucidate EA's impact on ureteral function, providing a robust scientific foundation for its clinical application.

Given the promising role of EA in first-stage RIRS, dedicated research efforts are warranted to validate its potential in enhancing UAS placement success, reducing

intraoperative ureteral trauma, and improving stone clearance rates. To address this, we propose expanding sample sizes in subsequent trials to strengthen evidence reliability.

This randomized, single-blind, placebo-controlled, multicenter clinical trial will enroll eligible participants to evaluate EA versus sham EA as an adjunct to first-stage RIRS. Key endpoints include UAS placement success rates, procedural safety (e.g., ureteral injury incidence), and stone-free outcomes, with the goal of establishing EA as a standardized adjunctive protocol.

2.Study object

The inclusion criteria for this study are explicitly defined as follows: (1) Voluntary participation in the trial with written informed consent provided and signed by the participant or their legal representative; (2) Age between 18 and 75 years (inclusive); (3) Confirmed diagnosis of upper ureteral or renal calculi (long-axis diameter ≥10 mm) via urinary tract CT imaging; (4) Scheduled for first-time ureteral stent placement.

Exclusion criteria are delineated in detail: (1) Patients with acute genitourinary infection or febrile conditions; (2) Individuals with a history of alpha-blocker use; (3) Those with solitary kidney, congenital urethral/ureteral malformations, or prior corrective surgeries; (4) Participants exhibiting acupuncture procedure-related phobia; (5) Enrollment in other clinical trials within the preceding three months; (6) Any additional conditions deemed by investigators to compromise trial integrity or participant safety.

3.Study design

This study is a randomized, single-blind, placebo-controlled, parallel-group, multicenter clinical trial with a superiority design, aimed at preliminarily evaluating the efficacy and safety of EA compared to sham EA in improving the success rate of UAS placement during first-stage RIRS. A total of 120 patients with urinary tract calculi requiring first-time ureteral stent placement under general anesthesia will be enrolled and rigorously randomized into the EA group or sham EA group. Comprehensive clinical data will be prospectively collected and analyzed to establish a robust scientific foundation for optimizing therapeutic strategies in urolithiasis management.

4.Sample size estimation

Based on preliminary trial results demonstrating a 90% success rate of UAS placement in the EA group compared to 75% in the sham EA group, this study adopts a conservative estimate that EA will yield at least a 15% superiority over sham EA for sample size calculation. With a two-sided significance level of 0.05, a 1:1 allocation ratio between groups, and 0.28 confidence interval width, a total of 110 participants (55 in the EA group and 55 in the sham EA group) are required to detect this superiority. Accounting for a potential 10% attrition rate, the adjusted sample size is 120 patients.

5.Interventions

5.1 Surgical and Anesthetic Method

Both the experimental and control groups underwent flexible ureteroscopy with ureteral stent placement in the operating room. Following lithotomy positioning,

participants received either EA or sham EA 10 minutes prior to general anesthesia induction, with stimulation maintained until procedure completion.

5.2 EA Protocol for the Experimental Group

In addition to standard general anesthesia, EA was administered 10 minutes preoperatively and sustained intraoperatively. Sterile acupuncture needles (Hwato brand, 0.25 mm × 40 mm) were inserted at Sanyinjiao (SP6), Zhaohai (KI6), and Taixi (KI3). After lifting, thrusting, and rotating the needles for 30 seconds, an electronic stimulator (SDZ-II model, Hwato brand) was connected, delivering continuous wave at 50 Hz with current intensity adjusted between 1–5 mA (maximal tolerable level without pain). Licensed acupuncturists with ≥5 years of experience performed all interventions following standardized training on acupoint localization and needling techniques. Should any acupuncture-related adverse events occur, the acupuncture needles will be immediately removed. The specific acupoint localization protocols are as follows:

Acupoint (international code)	Location	Angle and Depth
Sanyinjiao(SP6)	The medial side of the lower leg, 3 cun above the tip of the medial malleolus, posterior to the medial border of the tibia.	Insert the needle perpendicularly to the skin to a depth of 1-1.5 cun.
Zhaohai (KI6)	1 finger-width medial to the ankle joint.	Insert the needle perpendicularly to the skin to a depth of 1.5-2 cun.
Taixi (KI3)	2 finger-width medial to the ankle joint.	Insert the needle perpendicularly to the skin to a depth of 1-1.5 cun.

5.3 Sham EA Protocol for the Control Group

Participants in the control group will receive sham acupuncture following the same procedural timeline as the experimental group. Needles will be inserted 3 mm lateral to the verum acupoints in non-meridian, non-acupoint regions using superficial needling (1–4 mm penetration depth) without needle manipulation (lifting, thrusting, or

rotating) . Following sham needling, the needles will be connected to a deactivated electroacupuncture device with internally short-circuited wiring to prevent current output. Should any acupuncture-related adverse events occur, the needles will be immediately removed. Sham acupuncture will be performed by licensed acupuncturists with ≥5 years of experience, who will undergo standardized training in sham acupoint localization and needling techniques prior to trial commencement.

The comparative characteristics of the experimental and control groups, including their shared features and key distinctions, are summarized in the table below:

Program	Experimental Group	Control Group
Common Points		
Stimulated Point Area	Leg	
Number of Acupoints/Non-Acupoints	6	
Number of Needles	6	
Specifications of Needling Instruments	Length: 4 mm, Diameter: 0.25 mm	
Difference		
Types of Stimulated Points	Acupoint	Non-Acupoint
Location of Stimulated Points	Sanyinjiao (SP6), Zhaohai (KI6), Taixi (KI3)	3 mm lateral to the acupoints in the experimental group
Needling Depth	Approximately 10 mm-30 mm	1-4mm
Deqi Sensation	Yes	No
Actual Current Output	Yes	No

6.Clinical observation index

(1) Primary endpoint

The primary endpoint is the proportion of patients who achieved successful UAS placement and completed the first-stage RIRS without major intraoperative complications.

(2) Secondary endpoints

Secondary outcomes include: (1) Surgical duration, recorded in minutes from incision to closure; (2) UAS insertion resistance, measured with an IMADA50N push-pull gauge (calibrated monthly for accuracy); (3) Ureteral injury rade, assessed at UAS removal using the Post-Ureteroscopic Lesion Scale (PULS) by two blinded urologists, with a third resolving discrepancies; (4) Stone clearance rate, defined as the proportion of patients stone-free on CT two weeks post-surgery; and (5) AEs, tracked during surgery and for two weeks afterward, including acupuncture-related (e.g., bruising) and surgical (e.g., infection) events. These outcomes capture procedural efficiency, tissue safety, and recovery, with standardized tools and blinded assessments ensuring reliability. Training on PULS grading will be provided to assessors to maintain consistency.

References

[1] Zeng, Guohua et al. "Prevalence of kidney stones in China: an ultrasonography based cross-sectional study." BJU international vol. 120,1 (2017): 109-116.

[2] Jeong YB，Doo AR，Park HS，et al.Clinical significance of ureteral stent removal by flexible cystoscopy on pain and satisfaction in young males: a prospective randomised control trial.Urolithiasis，2016，44(4):367-370.

[3] Wang M, Liu W, Ge J, Liu S. The immunomodulatory mechanisms for acupuncture practice. Front Immunol. 2023 Apr 6;14:1147718.

[4] Mao JJ, Liou KT, Baser RE, et al. Effectiveness of Electroacupuncture or Auricular Acupuncture vs Usual Care for Chronic Musculoskeletal Pain Among Cancer Survivors: The PEACE Randomized Clinical Trial. JAMA Oncol. 2021;7(5):720-727.

[5] Hu L, Yang J, Liu T, et al. Hotspots and Trends in Research on Treating Pain with Electroacupuncture: A Bibliometric and Visualization Analysis from 1994 to 2022. J Pain Res. 2023;16:3673-3691.

[6] Shah S, Godhardt L, Spofford C. Acupuncture and Postoperative Pain Reduction. Curr Pain Headache Rep. 2022;26(6):453-458.

[7] Yuan W, Wang Q. Perioperative acupuncture medicine: a novel concept instead of acupuncture anesthesia. Chin Med J (Engl). 2019;132(6):707-715

[8] Zhan X, Zhai X, Chen X, et al. Application of Electroacupuncture-Assisted Local Anesthesia in Transperineal Prostate Biopsy. Journal of Clinical Urology. 2024;39(2):131-135, 140..
