# Supplementary material for: Efficacy of preoperative electroacupuncture for ureteral access sheath placement during first-stage flexible ureteroscopy in urolithiasis: a multicenter, randomized, single-blind, sham-controlled trial protocol
Source: BMC Urol. 2025 Dec 9;25:301. doi: 10.1186/s12894-025-01993-3 (PMC12690886; doi:10.1186/s12894-025-01993-3)
Supplement: Supplementary file 3 — Supplementary Material 3: S3 File. Study protocol approved by the ethics committee (in Chinese). [file 12894_2025_1993_MOESM3_ESM.pdf]

电针对一期输尿管软镜碎石术中输尿管通道鞘置入成功率的疗效评估：一项随机、单盲、假电针平行对照、多中心临床研究

主要研究者：翟新宇，上海中医药大学附属曙光医院 泌尿外科

电子邮件：zhaixinyu_2008@sina.com

地址：上海市浦东新区张衡路 528 号

版本：2.0

日期：2025 年 4 月 20 日

摘要

引文:

逆行肾内手术 （RIRS） 是尿路结石的主要治疗方法，成功的输尿管通路鞘 （UAS） 放置是关键步骤。初步研究表明，术前电针 （EA） 可以提高 UAS 放置的成功率，减少输尿管损伤，并提高结石清除率。该试验评估了 EA 作为原发性 RIRS 辅助治疗的有效性和安全性。

方法与分析:

这项多中心、随机、单盲、假对照试验将计划接受初次 RIRS 的上输尿管结石或肾结石 （≥10 mm） 的 120 名成年患者。参与者将被随机分配 （1：1） 接受术前 EA 加全身麻醉或假 EA 加全身麻醉，跨多个疗程。主要结果是在初次 RIRS 期间成功放置 UAS 的患者比例。次要结局包括手术时间、UAS 插入阻力、输尿管损伤 （输尿管镜后病变量表 [PULS]）、术后 2 周的结石清除率以及术后 2 周内的不良事件 （AE）。将使用意向性治疗原则分析数据。

讨论：

本研究是第一个调查电针在提高初次 RIRS 期间 UAS 放置成功率和安全性方面的疗效的随机对照试验。研究结果将提供高质量的证据，以支持在临床实践中使用电针作为辅助治疗。通过全面的多维评估，本研究证明了电针在尿石症治疗中更广泛应用的潜力。进一步严格设计的临床试验对于验证和完善这种有前途的治疗方法至关重要。

研究方案

1. 研究背景

1．输尿管软镜碎石手术是治疗泌尿系结石的主要手段，术中输尿管软镜通道鞘的成功置入是重要环节，寻找一种能有效提高置管成功率的方法尤为关键

泌尿系结石是泌尿外科的常见病，最近流行病学调查结果显示我国患病率为1.61% ~20.45%，总患病率达到了 7.54%，同时呈逐渐上升趋势[1]。很多患者需行腔内微创手术治疗[2]，包括输尿管硬镜、输尿管软镜、经皮肾镜手术、腹腔镜切开取石手术等。随着技术与设备的发展，输尿管软镜（retrograde interrenal surgery, RIRS）手术已经成为泌尿系结石治疗的一种主要手段。手术过程中输尿管软镜通道鞘(ureteral access sheath, UAS)的成功置入是 RIRS 术中第一步,也是重要的一环[3]。UAS 的成功置入是 RIRS 重要的环节,如何把握好 UAS 置入时的力度，减少输尿管损伤风险，是值得探讨的问题。研究发现，输尿管镜手术过程中，由于结石的梗阻引起输尿管肌肉的痉挛及患者本身的输尿管狭窄带来的径向扩张阻力是导致 UAS 置入失败的主要原因。置鞘时暴力操作会导致一定程度的输尿管损伤，甚至输尿管穿孔、撕脱、断裂[3]。但是缺少量化的数据，多大的力才是暴力不得而知。虽然有一些研究分析了影响置鞘阻力的一些危险因素[4-6]，但并未提出一期 RIRS 置鞘过程中，减少置鞘阻力和输尿管损伤的有效方案。也有研究表明，术前一周服用 α 受体阻滞剂能有效增加一期 RIRS 手术过程中

UAS 的置入成功率，但是结石患者就诊时常常伴有剧烈的疼痛不适，服用药物一周后再进行手术往往无法满足患者对于尽快解除痛苦的要求。因此，能否寻找一种既能尽快手术，又能增加一期 RIRS 术中 UAS 置入成功率的方法尤为关键。

2. 电针治疗方法有良好的松弛输尿管平滑肌的作用。但电针是否能增加一期 RIRS 术中 UAS 置管成功率的研究尚无报道

在临床应用中，针灸对多种疾病具有显著的疗效。它可以缓解疼痛，如头痛、颈肩腰腿痛、关节炎等；治疗神经系统疾病，如面瘫、中风后遗症、失眠等；调节消化系统功能，如胃痛、腹泻、便秘等；还对妇科、男科、五官科等疾病有一定的治疗作用。与现代医学相比，针灸具有独特的优势。它操作简便、成本低廉、副作用小，对一些慢性疾病和疑难病症往往能起到意想不到的效果。同时，针灸还可以与其他疗法如电刺激等相结合，提高治疗效果。

电针作为针灸与现代医学相结合的一种辅助疗法，对多种类型的肌肉痉挛、疼痛缓解具有显著的临床疗效[5-6]。研究发现，电针能从多方面对输尿管平滑肌起到松弛作用。其作用机制主要有以下几个方面：

一、神经调节机制

1. 刺激神经系统：电针通过刺激特定穴位，激活神经系统的感觉传入通路。这些感觉信号传入中枢神经系统后，引起神经反射，调节自主神经系统的功能。通过调节交感神经和副交感神经的平衡，影响输尿管平滑肌的紧张度。副交感神经兴

奋可使输尿管平滑肌松弛，促进尿液排出；而交感神经兴奋则可能导致输尿管平滑肌收缩。

2. 释放神经递质：电针刺激可以促使神经系统释放一些神经递质，如内啡肽、乙酰胆碱等。内啡肽具有镇痛和放松平滑肌的作用，可对输尿管平滑肌产生直接的松弛效应。乙酰胆碱在某些情况下也可以调节输尿管的收缩和松弛，针灸可能通过调节乙酰胆碱的释放来影响输尿管平滑肌的功能。

二、内分泌调节机制

1. 影响激素水平：电针可能通过调节内分泌系统，影响激素的分泌和释放，从而对输尿管平滑肌产生作用。如前列腺素、一氧化氮等在输尿管的功能调节中起着重要作用。针灸可能调节这些激素的合成和释放，改变输尿管平滑肌的紧张度。

2. 调节体液平衡：内分泌系统还参与调节体液平衡，这也可能间接影响输尿管的功能。通过调节肾脏对水和电解质的排泄，影响尿液的生成和排出，进而影响输尿管的压力和蠕动。

三、局部组织调节机制

1. 改善血液循环：电针可以促进局部血液循环，增加组织的氧气和营养供应，减少代谢产物的堆积。改善血液循环可以减轻局部组织的水肿和炎症，从而缓解输尿管平滑肌的痉挛。

2. 调节肌肉张力：电针可能直接作用于输尿管平滑肌，调节其肌肉张力。通过刺激特定穴位，可以引起局部肌肉的收缩和放松，从而影响输尿管平滑肌的紧张

度。这种调节可能是通过神经反射、体液调节或直接的机械刺激等多种途径实现的。

电针松弛输尿管平滑肌的作用机制是复杂的，涉及神经、内分泌和局部组织等多个方面的调节。目前，虽然对其作用机制的研究还在不断深入，但电针作为一种传统与现代相结合的中医疗法，在缓解输尿管平滑肌痉挛、促进尿液排出等方面已经显示出了一定的临床疗效。

3. 前期预实验发现电针在一期 RIRS 术中能有效增加 UAS 置入的成功率、减轻输尿管损伤、增加结石清除率，值得进一步深入研究

课题组前期预实验发现电针在一期 RIRS 术中能有效增加 UAS 置入的成功率、减轻输尿管损伤、增加结石清除率具有显著效果，这可能有几方面的原因。首先，提高 UAS 置入的成功率具有重要意义。UAS 的顺利置入是 RIRS 成功的关键步骤之一。电针通过其独特的作用机制，可能调节了输尿管的平滑肌张力，使其更加松弛，从而便于 UAS 的置入。这不仅可以减少手术时间和难度，还能降低手术风险，提高手术的安全性。其次，减轻输尿管损伤是电针的另一大优势。在手术过程中，输尿管容易受到损伤，这可能导致术后并发症的发生，如输尿管狭窄、尿漏等。电针的干预可能通过调节局部血液循环、减少炎症反应等方式，保护输尿管组织，降低损伤的风险。最后，增加结石清除率对于患者的康复至关重要。结石的残留可能导致复发和再次手术，给患者带来痛苦和经济负担。电针可

能通过促进输尿管蠕动、改善尿液引流等方式，帮助术中碎石排出，提高结石清除率。

深入研究电针在 RIRS 中的作用机制，可以为临床治疗提供新的思路和方法。可以进一步探索电针的最佳刺激参数、穴位选择等问题，以提高其疗效和安全性。同时，还可以结合现代医学技术，如影像学、生理学等，深入了解电针对输尿管功能的影响，为其临床应用提供更加科学的依据。

综上所诉，电针在一期 RIRS 术中的潜在价值值得我们投入更多的精力进行深入研究，以期为患者提供更加安全、有效的治疗方法。因此，我们计划扩大样本规模，以更深入地验证电针在一期 RIRS 术中 UAS 置入成功率、减少输尿管损伤、增加结石清除率的显著效果。

本研究是一项随机、单盲、安慰剂平行对照、多中心临床研究。在纳入符合纳排标准的受试者后，评价电针对比假电针对提高一期 RIRS 手术 UAS 置入的成功率和安全性的影响。

2. 研究对象

关于入组标准，我们明确规定了以下几点：

（1）自愿参加本试验，本人或其法定代理人签署书面知情同意书。

（2）年龄为 18-75 岁（包含 18 岁，75 岁）。

（3）泌尿系统 CT 检查确诊为输尿管上段结石或肾结石（长径≥10mm）。

（4）首次需行输尿管支架置入术。

至于排除标准，我们亦制定了以下详细规定：其一，处于泌尿生殖系统急性感染期或发热期的患者将被排除在外；其二，有α受体阻滞剂用药史亦不符合入组条件；其三，有孤立肾、尿道及输尿管畸形手术史的患者将被排除；其四，恐惧针刺操作者的患者同样不符合入组要求；其五，入选前 3 个月内参加其他临床试验者的患者亦将被排除；最后，研究者认为不宜参与本试验的其他情况者亦将被排除。

3. 研究设计

本研究是一项随机、单盲、安慰剂平行对照、多中心、优效设计的临床试验，旨在以安慰剂（假电针）为对照，初步评价电针对提高一期 RIRS 手术 UAS 置入的成功率及安全性。本研究将共纳入 120 例首次需行输尿管支架置入术的尿路结石患者，以全身麻醉为基础麻醉，严格按照随机原则将其分为电针组和假电针组。在研究过程中，我们将全面收集患者的相关临床资料，并进行深入的前瞻性分析，以期能够为尿路结石患者的治疗提供更为科学、合理的依据。

4. 样本量估计

根据前期预试验结果，手术完成时电针组和空白对照组 UAS 置管成功率分别为 90%和 75%，根据目前的试验设计，保守估计电针组至少较假电针组增加 15%，以此进行样本量估算。双侧显著性水平为 0.05，电针组和假电针组比例为 1:1，则样本量需 110 例能以 0.28 的置信区间宽度识别出电针组优效于假电针组，分别是试验组（电针组）55 例和对照组（假电针组）55 例，另考虑 10%脱落率，样本量最终则为 120 例。

5. 干预措施

5.1 手术、麻醉操作方式

试验组和对照组均在手术室内行输尿管软镜下输尿管支架置入术，两组患者摆截石位体位后，按照分配结果在全麻开始前 10 分钟进行电针或假电针治疗，电针/假电针持续至手术完成。

5.2 试验组电针操作方式

试验组在常规给予全身麻醉的基础上，在术前 10min 行电针刺激并留针至手术结束。采用无菌针灸针（华佗牌，规格：0.25mm*40mm）对患者三阴交、照海、太溪穴进行针刺和捻转提插 30s 后接入电子针疗仪（SDZ-II 型，华佗牌），采用连续波 50HZ，电流强度在 1-5mA（最好为患者无疼痛感条件下所能耐受的最大程度）。针刺由具有至少 5 年经验的固定执业针灸师实施。针灸师在试验开始前均接受了穴位和针刺操作方面的培训，并记录干预完成情况。如患者出现任何与针灸相关的不良事件，将拔取针灸针。取穴定位具体如下：

穴位（国际编码）	定位	针刺角度及深度
三阴交（SP6）	小腿内侧，足内踝尖上 3 寸，胫骨内侧缘后方	垂直于皮肤，进针 1-1.5 寸
照海（RN4）	踝关节内侧 1 指	垂直于皮肤，进针 1.5-2 寸
太溪（SP6）	踝关节上方 2 指	垂直于皮肤，进针 1-1.5 寸

注：1 寸≈25mm

5.3 对照组假电针操作方式

对照组患者进行假针刺，对照组患者干预流程与试验组一致，但针刺部位位于试验组穴位旁开 3mm 非经非穴处，针刺方法采用浅针刺法，仅透皮 1-4mm，行假针刺后，不进行提插捻转法，假针刺后，接入内部线路短路的电针仪器，无实际电流输出。如患者出现任何与针灸相关的不良事件，将拔取针灸针。假针刺由具有至少 5 年经验的固定执业针灸师实施。针灸师在试验开始前均接受了假穴位和假针刺操作方面的培训，并记录干预完成情况。

试验组与对照组的共同点和区别点如下表所示：

项目	试验组	对照组
相同点		
受刺激点区域	腿部	
穴数/非穴数	6	
针数	6	
针具规格	长度：40mm，直径：0.25mm	
差异		
刺激点的种类	穴位	非穴位
刺激点的位置	三阴交、照海、太溪	试验组穴位旁开 3mm
针刺深度	大约 10mm-30mm	1-4mm
是否得气	是	否
是否有实际电流输出	是	否

6. 临床观察指标

（1）主要终点

主要终点是一期输尿管软镜碎石术 UAS 成功置入并顺利完成手术的患者比例。

（2）次要终点

次要终点包括（1）手术时间；（2）手术过程中 UAS 置入时的置鞘阻力（IMADA50N 推拉力计）；（3）手术完成时退出 UAS 时观察输尿管的损伤程度（按照输尿管损伤量表（post-ureteroscopiclesionscale, PULS）进行分级；

（4）手术完成后 2 周的结石清除率；（5）手术过程中及手术后 2 周内的不良事件

参考文献

[1] Zeng, Guohua et al. “Prevalence of kidney stones in China: an ultrasonography based cross-sectional study.” BJU international vol. 120,1 (2017): 109-116.

[2] Jeong YB，Doo AR，Park HS，et al.Clinical significance of ureteral stent removal by flexible cystoscopy on pain and satisfaction in young males: a prospective randomised control trial.Urolithiasis，2016，44(4):367-370.

[3] Wang M, Liu W, Ge J, Liu S. The immunomodulatory mechanisms for acupuncture practice. Front Immunol. 2023 Apr 6;14:1147718.

[4] Mao JJ, Liou KT, Baser RE, et al. Effectiveness of Electroacupuncture or Auricular Acupuncture vs Usual Care for Chronic Musculoskeletal Pain Among Cancer Survivors: The PEACE Randomized Clinical Trial. JAMA Oncol. 2021;7(5):720-727.

[5] Hu L, Yang J, Liu T, et al. Hotspots and Trends in Research on Treating Pain with Electroacupuncture: A Bibliometric and Visualization Analysis from 1994 to 2022. J Pain Res. 2023;16:3673-3691.

[6] Shah S, Godhardt L, Spofford C. Acupuncture and Postoperative Pain Reduction. Curr Pain Headache Rep. 2022;26(6):453-458.

[7] Yuan W, Wang Q. Perioperative acupuncture medicine: a novel concept instead of acupuncture anesthesia. Chin Med J (Engl). 2019;132(6):707-715

[8] 占向阳,翟新宇,陈杏林等.电针辅助局部麻醉在经会阴前列腺穿刺活检术中的应用[J].临床泌尿外科杂志,2024,39(02):131-135+140.
